# Predicting Re-Institutionalization in a Sample of Older Adults With Severe Mental Illness

**DOI:** 10.1093/geroni/igaf122.3828

**Published:** 2025-12-31

**Authors:** Deborah Finkel, Gavin Green, Marie Ernsth-Bravell, Pia Bülow, Per Bülow

**Affiliations:** University of Southern California, Los Angeles, California, United States; Utah State University, Logan, Utah, United States; Jönköping University, Jönköping, Jonkopings Lan, Sweden; School of Health and Welfare, Jönköping, Jonkopings Lan, Sweden; School of Health and Welfare, Jönköping, Jonkopings Lan, Sweden

## Abstract

After the Swedish Mental Health Care reform enacted in 1995, many individuals with severe mental illnesses (SMI) were released from long-term care institutions. Many older adults with SMI experienced most of their care during an era of care ideology characterized as that of the total institution, which meant longer periods of institutionalization. Managing new living and support situations challenged many older adults with SMI and as a result, many experienced re-institutionalization. The aim of the current analyses was to identify the older adults with SMI who were re-institutionalized after the 1995 care reforms. We used data from surveys collected in 1996, 2001, 2006, and 2011 from 441 older adults with SMI in a region in southern Sweden (mean age in 1996 = 61.2, SD = 11.4; 54.6% female), and data from national registers. The sample was divided into 3 groups: remained in their own home (N = 251), moved from their home to an institution (N = 37), and remained in an institution (N = 153). Logistic regression indicated that total years of institutionalization and current level of functioning distinguished all 3 groups. Compared with group 1, adults in group 2 were older, were younger at first admission to a mental hospital, had more total years of institutionalization, more needs assessed by the Camberwell Assessment of Needs, and less education. Results reveal the long-term consequences of lengthy institutionalization, including greater risk of developing “institutional syndrome” that can inhibit ability to adjust to living in the community, particularly for adults also experiencing the challenges of aging.

